# Clinical pre-test probability for obstructive coronary artery disease: insights from the European DISCHARGE pilot study

**DOI:** 10.1007/s00330-020-07175-z

**Published:** 2020-09-09

**Authors:** Sarah Feger, Paolo Ibes, Adriane E. Napp, Alexander Lembcke, Michael Laule, Henryk Dreger, Björn Bokelmann, Gershan K. Davis, Giles Roditi, Ignacio Diez, Stephen Schröder, Fabian Plank, Pal Maurovich-Horvat, Radosav Vidakovic, Josef Veselka, Malgorzata Ilnicka-Suckiel, Andrejs Erglis, Teodora Benedek, José Rodriguez-Palomares, Luca Saba, Klaus F. Kofoed, Matthias Gutberlet, Filip Ađić, Mikko Pietilä, Rita Faria, Audrone Vaitiekiene, Jonathan D. Dodd, Patrick Donnelly, Marco Francone, Cezary Kepka, Balazs Ruzsics, Jacqueline Müller-Nordhorn, Peter Schlattmann, Marc Dewey

**Affiliations:** 1 Charité – Universitätsmedizin Berlin, Humboldt-Universität and Freie Universität zu Berlin, Berlin, Germany; 2 Department of Cardiology, Aintree University Hospital, Liverpool, UK; 3 University of Central Lancashire, Liverpool, UK; 4 Institute of Cardiovascular and Medical Sciences, Glasgow University, Glasgow, UK; 5 Department of Cardiology, Basurto University Hospital Bilbao, Bilbao, Spain; 6 Department of Cardiology, ALB FILS KLINIKEN, Goeppingen, Germany; 7 Department of Radiology and Department of Cardiology, Medical University Innsbruck, Innsbruck, Austria; 8 Department of Radiology, Medical Imaging Centre, Semmelweis University, Budapest, Hungary; 9 Department of Cardiology, Clinical Hospital Center “Zemun”, Faculty of Medicine, University of Belgrade, Zemun, Belgrade Serbia; 10 Department of Cardiology, Motol University Hospital and 2nd School of Medicine, Charles University, Prague, Czech Republic; 11 Department of Cardiology, Wojewodzki Szpital Specjalistyczny We Wroclawiu, Wrocław, Poland; 12 Latvian Centre of Cardiology, Pauls Stradins Clinical University Hospital, Riga, Latvia; 13 Department of Cardiology, Cardio Med Medical Center Targu-Mures, Târgu Mureș, Romania; 14 Hospital Universitari Vall d´Hebron, Department of Cardiology. Vall d’Hebron Institut de Recerca (VHIR). Universitat Autònoma de Barcelona, Barcelona, Spain; 15 Centro de Investigación Biomédica en Red-CV, CIBER CV, Barcelona, Spain; 16 Department of Radiology, Azienda Ospedaliero Universitaria di Cagliari, Cagliari, Italy; 17 Department of Cardiology and Radiology, Rigshospitalet, University of Copenhagen, Copenhagen, Denmark; 18 Department of Diagnostic and Interventional Radiology, UNIVERSITY LEIPZIG –Heart Center Leipzig, Leipzig, Germany; 19 Department of Cardiology, Institute for Cardiovascular Diseases of Vojvodina, Faculty of Medicine, University of Novi Sad, Novi Sad, Serbia; 20 Turku PET Centre and Heart Centre, Turku University Hospital, Turku, Finland; 21 Department of Cardiology, Centro Hospitalar de Vila Nova de Gaia, Vila Nova de Gaia, Portugal; 22 Department of Cardiology, Lithuanian University of Health Sciences, Kaunas, Lithuania; 23 Department of Radiology, St. Vincent’s University hospital, School of Medicine, University College Dublin, Dublin, Ireland; 24 Department of Cardiology, Southeastern Health and Social Care Trust, Belfast, Ireland; 25 Department of Radiological, Pathological and Oncological Sciences, Sapienza University of Rome, Rome, Italy; 26 Dept. of Coronary and Structural Heart Diseases, Institute of Cardiology, Warsaw, Poland; 27 Royal Liverpool and Broadgreen University Hospital, Liverpool, UK; 28 Berlin School of Public Health Berlin, Berlin, Germany; 29 Institut für Statistik, Medizinische Informatik, Datenwissenschaften Universitätsklinikum Jena, Leipzig, Germany; 30 Charité—Universitätsmedizin Berlin Department of Radiology, Berlin Institute of Health, Berlin, Germany; 31 DZHK (German Centre for Cardiovascular Research), partner site Berlin, Germany

**Keywords:** Coronary artery disease, Computed tomography angiography, Probability of disease, Prevalence

## Abstract

**Objectives:**

To test the accuracy of clinical pre-test probability (PTP) for prediction of obstructive coronary artery disease (CAD) in a pan-European setting.

**Methods:**

Patients with suspected CAD and stable chest pain who were clinically referred for invasive coronary angiography (ICA) or computed tomography (CT) were included by clinical sites participating in the pilot study of the European multi-centre DISCHARGE trial. PTP of CAD was determined using the Diamond-Forrester (D+F) prediction model initially introduced in 1979 and the updated D+F model from 2011. Obstructive coronary artery disease (CAD) was defined by one at least 50% diameter coronary stenosis by both CT and ICA.

**Results:**

In total, 1440 patients (654 female, 786 male) were included at 25 clinical sites from May 2014 until July 2017. Of these patients, 725 underwent CT, while 715 underwent ICA. Both prediction models overestimated the prevalence of obstructive CAD (31.7%, 456 of 1440 patients, PTP: initial D+F 58.9% (28.1–90.6%), updated D+F 47.3% (34.2–59.9%), both *p* < 0.001), but overestimation of disease prevalence was higher for the initial D+F (*p* < 0.001). The discriminative ability was higher for the updated D+F 2011 (AUC of 0.73 95% confidence interval [CI] 0.70–0.76 versus AUC of 0.70 CI 0.67–0.73 for the initial D+F; *p* < 0.001; odds ratio (or) 1.55 CI 1.29–1.86, net reclassification index 0.11 CI 0.05–0.16, *p* < 0.001).

**Conclusions:**

Clinical PTP calculation using the initial and updated D+F prediction models relevantly overestimates the actual prevalence of obstructive CAD in patients with stable chest pain clinically referred for ICA and CT suggesting that further refinements to improve clinical decision-making are needed.

**Trial registration:**

https://www.clinicaltrials.gov/ct2/show/NCT02400229

**Key Points:**

*• Clinical pre-test probability calculation using the initial and updated D+F model overestimates the prevalence of obstructive CAD identified by ICA and CT.*

*• Overestimation of disease prevalence is higher for the initial D+F compared with the updated D+F.*

*• Diagnostic accuracy of PTP assessment varies strongly between different clinical sites throughout Europe.*

**Electronic supplementary material:**

The online version of this article (10.1007/s00330-020-07175-z) contains supplementary material, which is available to authorized users.

## Introduction

In clinical routine, coronary artery disease (CAD) can be diagnosed by using invasive and non-invasive diagnostic tests. Choosing the appropriate and most beneficial diagnostic test for each patient is highly important, [1] since invasive and non-invasive tests have different advantages and disadvantages. Invasive coronary angiography (ICA) enables invasive therapeutic measures in the same session, but is associated with a small risk of procedural complications [2, 3]. These risks can be avoided in clinical practice by using non-invasive tests instead [4]. Coronary computed tomography (CT) angiography is a safe non-invasive diagnostic test [5] with a very high negative predictive value, making it especially valuable for ruling out obstructive CAD [6–8]. The calculation of pre-test probability (PTP) of disease can facilitate selecting patients with stable angina pectoris for the most beneficial diagnostic procedure [9]. The current European Guidelines recommend the selection of the appropriate diagnostic test according to the individual patient’s CAD probability [10, 11]. Thus, the correct assessment of the likelihood of CAD is important to select patients for the diagnostic procedure with the highest expected clinical benefit [12]. Multiple PTP calculators exist and have been established in clinical practice. The Diamond-Forrester (D+F) PTP calculation model, which is recommended by the current European guidelines for patients with stable chest pain, was introduced as early as 1979 (initial D+F) and is based on three clinical parameters that have to be assessed in patients (the patient’s age, the type of angina presentation, and the patient’s gender) [13]. Studies have shown that the original D+F model overestimated the prevalence of CAD [14, 15]. The original D+F was statistically updated by Genders et al in 2011 to improve the prediction of obstructive CAD especially in women and patients with atypical angina presentation (updated D+F 2011) being clinically referred to ICA [16]. The purpose of this study was to analyse the accuracy of PTP calculation of CAD using the original and updated D+F prediction models in patients with stable chest pain with a clinical referral for either CT or ICA in a multi-centre pan-European setting [17].

## Materials and methods

### Study design and patient recruitment

This study was performed as part of the pilot study of the European multi-Diagnostic Imaging Strategies for Patients With Stable Chest Pain and Intermediate Risk of Coronary Artery Disease’ (DISCHARGE) trial (www.dischargetrial.eu; FP7 2007-2013, EC-GA 603266; trial registration https://www.clinicaltrials.gov/ct2/show/NCT02400229) [18]. The DISCHARGE trial compares the effectiveness of invasive versus non-invasive coronary angiography. Patients were prospectively included by 25 clinical sites across Europe from 16 European countries.

The DISCHARGE pilot study was conducted in patients clinically referred for either CT or ICA. Both examinations were performed at the Department of Radiology/Cardiology at each of the 25 clinical sites. The decision for either diagnostic test was made in an outpatient setting, and the patients were then clinically referred to elective CT or ICA. Before allocation to the subsequent clinical test, the clinical parameters for PTP calculation were assessed in all patients. The complete recruitment period was about 3 years, extending from April 2014 to July 2017. The mean recruitment period per clinical site was 8 months (8.1 ± 4.8 months), ranging from 1 to 17 months.

### Inclusion criteria

Inclusion and exclusion criteria were relatively broad. Patients were eligible for inclusion into the non-randomised DISCHARGE pilot study if they had a routinely scheduled clinical examination for suspected CAD and stable chest discomfort/chest pain and were at least 30 years of age. Patients with known CAD including a history of prior myocardial infarction or revascularization were not included. Patients were excluded from the study if they were not in sinus rhythm, were pregnant, or required haemodialysis.

### Study outcomes

The purpose of the pilot study was to acquire information on the routine clinical practice of CTA and ICA at different clinical sites. In this paper, we investigate the accuracy of PTP calculation of obstructive CAD using the Diamond-Forrester (D+F) prediction models in patients clinically referred to CT or ICA. Each of the 25 clinical sites planned to include at least 60 patients routinely scheduled for the examinations (30 CTA and 30 ICA).

### Ethics

The ethics approval for the main DISCHARGE trial (EA1/294/13) included the pilot study. Depending on local site requirements for data acquisition, written and/or oral informed consent was obtained from all patients participating in the DISCHARGE pilot study. The study participants did not undergo any follow-up examinations or investigational procedures. The DISCHARGE project is funded by the EU-FP7 Framework Programme (FP7 2007-2013, EC-GA 603266, EC-GA 603266) but the clinical sites did not receive any funding for the pilot study which was an own contribution by all. Only research staff at the coordinator site received funding for coordinating the pilot study.

### Study conduct

The ICA and CT examinations had to be performed according to local standards. Vendor-specific CT protocols and a 10-step guide for CT and ICA were finalised by the DISCHARGE consortium and distributed to all involved staff members at all clinical sites. In addition, we performed two cardiac CT workshops in Berlin for the physicians involved in the examinations at all clinical sites to create a comparable basis of CT reading abilities for all participating centres [19].

### Assessment of pre-test probabilities

**Fig. 1:**
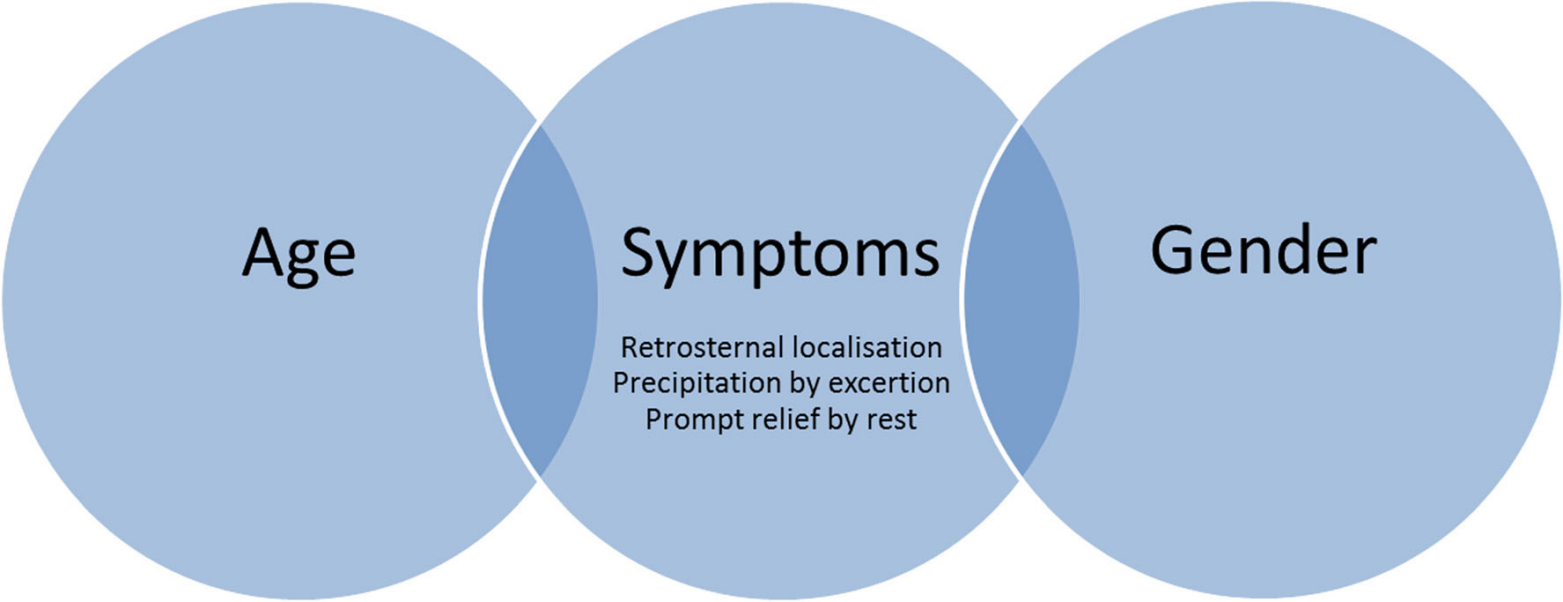
Three clinical parameters for pre-test probability estimation

Patients were included independently of their estimated or calculated disease probability. PTP of all included patients were assessed by comparing the initial D+F model introduced in 1979 and the updated D+F model from 2011 [13, 16]. Both models of pre-test calculation of CAD probability include only three clinical parameters that had to be assessed in all patients: the patient’s age, the type of angina presentation, and the patient’s gender (Fig. 1) [20]. The typicality of angina presentation was based on the presence of the following three criteria: retrosternal localisation of pain, precipitation by exertion, and prompt relief by rest or after nitroglycerin. [20] Dependent on how many criteria were fulfilled patients were allocated to one of the following categories: typical angina (all 3 criteria present), atypical angina (2 of 3 criteria), non-anginal chest discomfort (1 of 3 criteria) or other chest discomfort (no criteria fulfilled). PTP for all patients at all sites was calculated by the coordinating site based on the information provided in the CRF.

### Assessment of obstructive CAD

Obstructive CAD was defined by ICA and CT as the presence of at least one 50% coronary artery diameter stenosis on a per-patient level. As CT has a high sensitivity/specificity for the detection of significant CAD, we used CT results equally as ICA results in this study [6]. If ICA was performed after clinically indicated CT, the ICA result was selected for the final analysis of the accuracy of PTP, since ICA is the reference standard for the diagnosis of CAD. All examinations were evaluated locally at each clinical site by qualified and trained readers for CT and cardiologists for ICA according to their local standard of care, e.g. by using quantitative coronary analysis.

### Statistical analysis

Values are given as arithmetic mean (standard deviation), as median (interquartile range; IQR), or as number of patients (percentages). We performed the statistical analysis by using SPSS version 20 and R 3.4.4 [21]. A *p* value of ≤ 0.05 was defined to indicate statistical significance. The dependent *t* test for paired samples was used for the comparison of continuous variables. To compare pared dichotomous variables, we performed McNemar’s test. We used the area under the receiver-operating-characteristic curve (AUC) to compare the discriminative power of the two pre-test prediction models. To compare the two models in terms of their diagnostic accuracy, we performed a logistic regression analysis with the outcome (CAD or no CAD) as a dependent variable and the respective PTP and the method of computation as a predictor. In order to take care of the variability between sites, we applied a random intercept for site and correlation between observations. In addition, we performed a net reclassification analysis using 50% as a cutoff value for the PTP.

## Results

### Study population

**Fig. 2:**
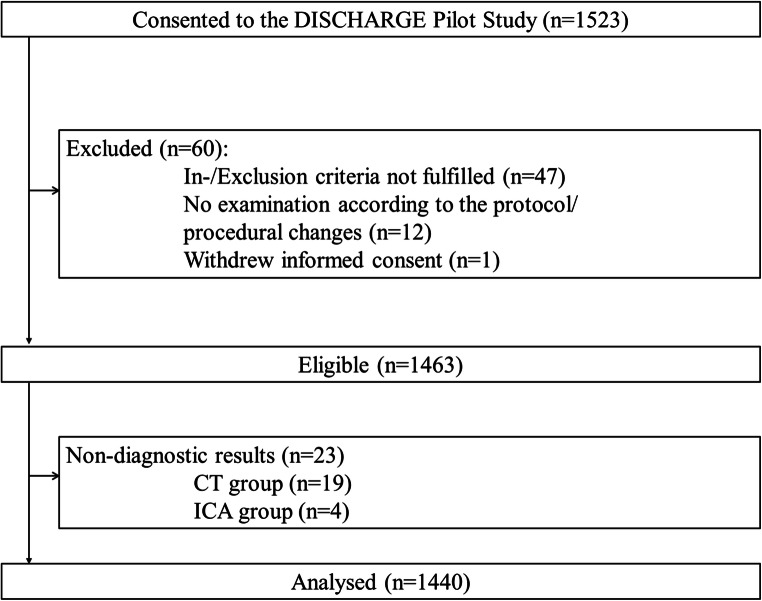
Flowchart of the pilot study. A total of 1463 patients were eligible for study inclusion. For the final analysis, we included 1440 patients (with 654 female and 786 male patients)

It was planned to include 1523 patients (see flowchart in Fig. 2 for further details). Sixty of these patients were retrospectively excluded. The most frequent reason was a lack of adherence to the inclusion and exclusion criteria. Thus, 1463 eligible patients were included in the study and underwent CT or ICA according to clinical referral. Nevertheless, 23 patients had non-diagnostic examinations and were excluded. For the final analysis, 1440 patients were included (654 female and 786 male patients, 45% and 55%, respectively). In this final population, 725 patients underwent CT (358 female, 367 male) and 715 underwent ICA (296 female, 419 male).

### Patient characteristics

The number of patients with typical chest pain was higher in the ICA group than in the CT group with 53% (378 of 715) versus 35% (253 of 725; Table 1). There were more patients with atypical angina/non-anginal chest pain or other chest discomfort in the CT group (65%) compared with those in the ICA group (47%). The mean age of patients was 64 years in the ICA group (63.8 ± 9.7 years; Table 1) and 59 years in the CT group (58.7 ± 11.3 years) with an overall range of 30–86 years. The male-to-female ratio was slightly higher in the ICA group with 59% male patients (419 male and 296 female patients) than in the CT group with 51% male patients (367 male and 358 female patients).

**Table 1:** Distribution of clinical parameters in the ICA and CT group

		ICA group (*N* = 715)		CT group (*N* = 725)	
Symptoms	Typical angina pectoris	378	(52.9%)	253	(34.9%) *
	Atypical angina pectoris	166	(23.2%)	247	(34.1%) *
	Non-anginal chest discomfort	142	(19.9%)	194	(26.8%) *
	Other chest discomfort	29	(4.1%)	31	(4.3%)
Gender	Female	296	(41.4%)	358	(49.3%)
	Male	419	(58.6%)	367	(50.6%)
Age	Median (IQR), year	64	(57–72)	58	(51–67)

The number of patients with typical chest pain was higher in the ICA group than in the CT group (53% versus 35%). More patients in the CT group presented with atypical angina or non-anginal chest pain than in the ICA group (61% versus 43%). The mean age of patients was higher in the ICA group than in the CT group (64 versus 59 years). The male-to-female ratio was slightly higher in the ICA group compared with that in the CT group. *Statistical significance

### Examination results and accuracy of pre-test probability prediction

**Fig. 3:**
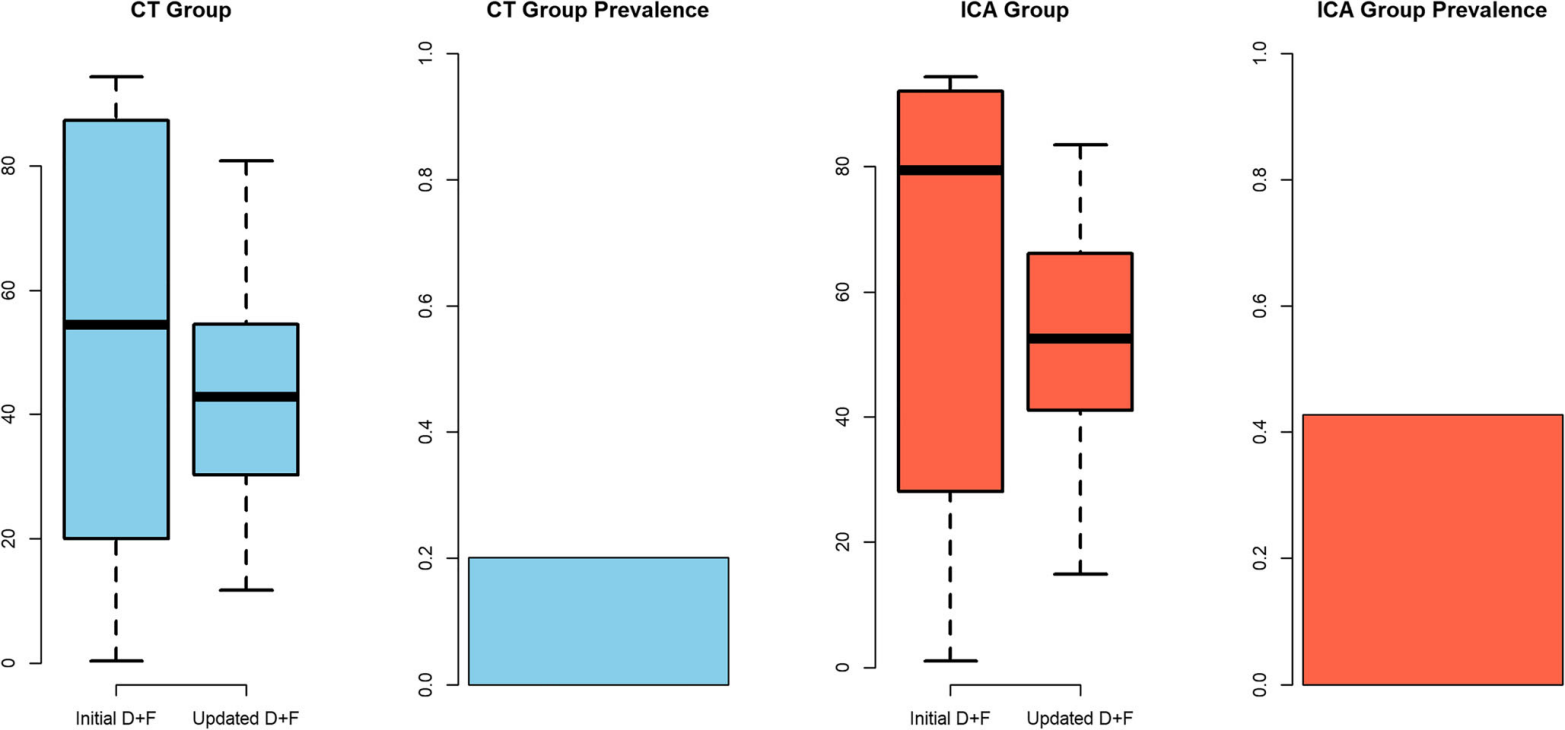
Pre-test probability and coronary artery disease (CAD) prevalence. A total of 725 patients underwent CT, and 715 underwent ICA. The average prevalence of obstructive CAD in the entire population was 32% with a higher prevalence in the ICA group (45%) versus the CT group (19%). The actual disease probability was relevantly overestimated by both prediction models with a higher overestimation of disease prevalence for the initial version compared with the updated D+F (*p* < 0.001). This overestimation again was higher in the CT group compared with that in the ICA group (*p* < 0.001)

The overall prevalence of obstructive CAD was 31.7% (456 of 1441 patients; Figure 3). In detail, the prevalence was higher in the ICA group (45.1%; 322 of 715 patients) compared with the CT group (18.5%; 134 of 725 patients; *p* < 0.001), and the overall prevalence ranged between 15.3% and 49.2% among the 25 clinical sites (Supplementary material, Online Figure 1). In the ICA group, the prevalence at the individual clinical sites ranged between 23.3% and 76.7%, and between 3.4% and 34.5% in the CT group.

The prevalence of obstructive CAD identified by ICA and CT (31.7%, 456 of 1440 patients) was overestimated by both prediction models (PTP: initial D+F 58.9% (28.1–90.6%) and updated D+F 47.3% (34.2–59.9%), both *p* < 0.001; Fig. 3). Comparison of both prediction models showed overestimation of CAD to be slightly less for the updated D+F compared with the initial version (*p* < 0.001). The PTP calculated with the initial D+F version was 51% in the CT group (median 54.4% (18.6–79.4%)) versus 65% in the ICA group (median 69.7% (32.4–92%)). In both groups, the PTP of CAD was significantly lower when calculated with the updated D+F version: 43% in the CT group (median 41.4% (29.8–53.2%)) and 53% in the ICA group (median 51.8% (40.6–65.4%)). Thus, both pre-test calculators overestimated the actual prevalence of obstructive CAD of 45% in the ICA group and 19% in the CT group (*p* < 0.001). The overestimation was higher for the initial D+F calculation compared with the updated D+F version in both the CT and the ICA group (*p* < 0.001) and was higher in the CT group compared with that in the ICA group (*p* < 0.001) for both prediction models.

### Discriminative ability and NET reclassification analysis

**Fig. 4:**
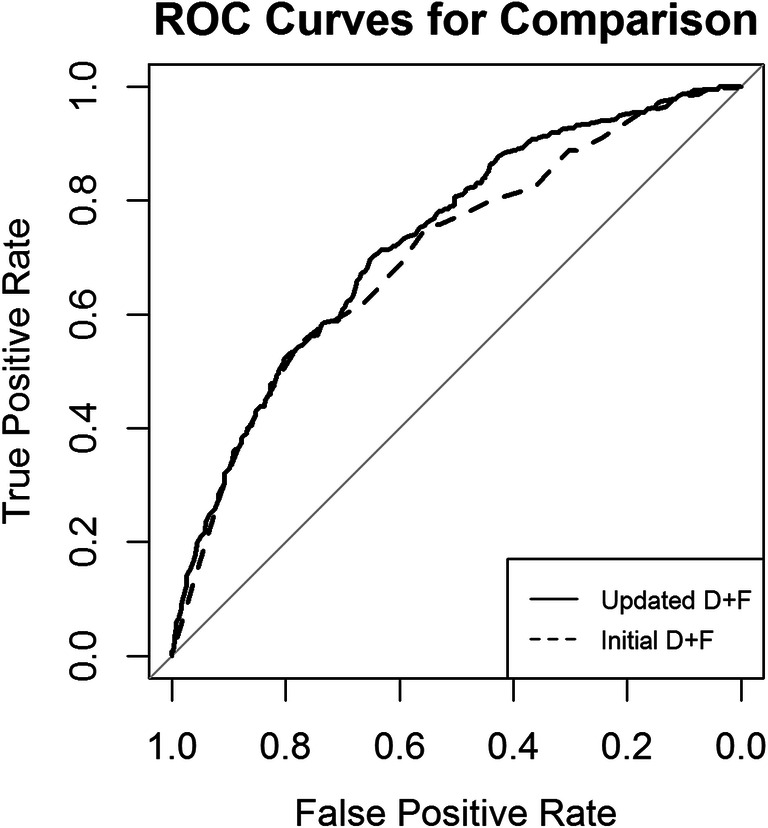
Comparison of the discriminative power of pre-test probability prediction models. The discriminative ability was higher for the updated D+F 2011 compared with the initial D+F prediction model. The AUC, which is a parameter of the discriminative ability, was 0.73 for the updated D+F (AUC; 95% confidence [CI] interval 0.70–0.76). The initial D+F had an AUC of 0.70 (95% CI 0.67–0.73; *p* < 0.001)

The discriminative ability was stronger for the updated D+F 2011 with an area under the receiver operating curve of 0.73 (AUC; 95% confidence interval [CI] 0.70–0.76; Fig. 4) compared with an AUC of 0.70 (CI 0.67–0.73) for the initial D+F prediction model (*p* < 0.001). The discriminative ability differed between the 25 clinical sites (Supplementary material, Online Figure 2). The logistic regression analysis with random intercept for the site showed a significant effect of the method with a coefficient equal to 0.44. This corresponds to an odds ratio of 1.55 (95% CI 1.29–1.86; Table 2). Thus, this result is in favour of the updated D+F model. There is also substantial variability between sites measured by the variance of the random intercepts with a value of 0.14.

**Table 2:** Logistic regression analysis with random effects

Random effects				
Groups name	Variance	Standard deviation		
Clinical site (intercept)	0.1371	0.3703		
Number of observations, 2880; groups: site, 25				
Fixed effects				
	Estimate	Standard error	*Z* value	Pr (>|*z*|)
(Intercept)	− 2.734	0.16884	− 16.192	< 2e–16***
Pre-test probability	3.04813	0.19998	15.243	< 2e–16***
Method (original D+F or updated D+F 2011)	0.43833*	0.09312	4.707	2.51e–06***
Correlation of fixed effects				
	(Intercept)	Pre-test probability		
Pre-test probability	− 0.818			
Method (original D+F or updated D+F 2011)	− 0.566	0.392		

We performed a logistic regression analysis with the outcome (prevalence; CAD or no CAD) as dependent and the respective pre-test probability and the method of computation as predictors using the stacked data set (method original D+F versus updated D+F 2011). In order to take care of the variability between sites, we applied a random intercept for site. There is a significant effect of the method with a coefficient equal to 0.44, corresponding to an odds ratio of 1.55 (95% CI 1.29, 1.86). Significance codes: 0 = ‘***’, 0.001 = ‘**’, 0.01 = ‘*’, 0.05 = ‘.’ 0.1 = ‘ ’ 1; *Statistical significance

As a result of the NET reclassification analysis, there is an improvement due to the updated D+F model (NRI categorical 0.11 95% CI (0.05–0.16); *p* < 0.001; Table 3). Thus, using a cutoff value of 50% PTP for CAD, the updated D+F model particularly reclassifies patients without CAD but with a PTP of ≥ 50% to a PTP < 50% compared with the initial D+F model.

**Table 3:** NET reclassification index

		Updated D+F 2011 (*n*)		% Reclassified
Outcome: no CAD (*n* = 984)				
	Pre-test probability	< 50%	≥ 50%	
Initial D+F	< 50%	381	41	10
	≥ 50%	266	296	47
Outcome: CAD (*n* = 456)				
	Pre-test probability	< 50%	≥ 50%	
Initial D+F	< 50%	66	23	26
	≥ 50%	79	288	22
Combined data				
	Pre-test probability	< 50%	≥ 50%	
Initial D+F	< 50%	477	64	13
	≥ 50%	345	584	37

NRI (categorical) [95% CI], 0.1059 [0.0532–0.1585]; *p* value, 0.00008

Net reclassification analysis using 50% as a cutoff value for the pre-test probability. As a result, it turns out that there is an improvement due to the Genders model (NRI categorical 0.11 95% CI (0.05–0.16); *p* < 0.001); *n* number of patients

## Discussion

The results of this multi-centre European study show the following:

1. The PTP calculated by the D+F model (initial and statistically updated version) relevantly overestimates the actual prevalence of CAD in patients clinically referred for ICA and CT with stable chest pain.
2. The updated D+F performs slightly better than the initial D+F.
3. Overestimation is higher in patients clinically referred to CT than in those clinically referred to ICA.
4. There is tremendous variability in the diagnostic accuracy of PTP assessment between different clinical sites which were trained in the basic concept of PTP assessment and evaluation of chest pain type.

### Interpretation of the results in the clinical context

In this multi-centre clinical trial, we compared the CAD PTP estimation of the initial D+F with the updated D+F in a non-selective pragmatic cohort of both patients referred on clinical grounds to CTA as well as ICA. Our trial shows that the statistically updated D+F has a slightly higher discriminative ability and therefore tends to estimate the CAD probability more accurately than the initial D+F [13, 16]. For patients routinely scheduled for ICA, the clinical estimation of PTP was more exact than for patients referred for CTA. Possibly, the reason is not primarily the diagnostic test itself for which the patients are scheduled, but the clinical presentation which resulted in the decision to refer the patients for either diagnostic test [22]. In our patient population, the prevalence was higher for the patients scheduled for ICA than for the CT patients. In this non-randomised study setting, this is logical from the clinical point of view, since ICA offers the possibility of subsequent treatment with angioplasty and coronary stenting.

Our results show variable prevalence of obstructive CAD in patients routinely scheduled for ICA and CTA for the 25 clinical sites included in our analysis. Since the prevalence of disease influences the accuracy of the applied clinical tests, it is highly important to know the local disease prevalence [10]. As our study sites are spread across Europe, multiple factors affect the local disease prevalence and discriminative ability. Overestimation of PTP of CAD will lead subsequently to increased downstream diagnostic testing which increases the possibility of adverse events and costs for the health care system. As recent studies have shown [23], the prevalence of obstructive CAD is relatively low in patients electively referred to ICA/CT to evaluate stable chest pain.

### Comparison with other studies

This study includes a large-scale prospective European cohort with both CTA and ICA being the combined gold standard in a non-specified patient cohort. Recent scientific data on the clinical application of PTP calculation in patients with suspected CAD are rare.

There are only few other papers that have assessed the accuracy of the D+F model to patients referred for CTA. They all have in common to include only patients with low PTP being clinically assigned to CT but do not compare with patients clinically referred to ICA. A study of Wasfy et al [24] was in an American cohort including patients being referred only for CTA based on a low PTP. A more recent study evaluated 3 scores among patients with suspected CAD in the CTA randomised arm of the SCOT-HEART study for the outcome of obstructive CAD by coronary CTA [25]: the modified D+F, CAD Consortium clinical score (CAD2), and CONFIRM risk score (CRS). They found that the best calibrator of obstructive CAD was the updated D+F, which goes in line with the results of our study. Another study has shown PTP calculation according to D+F to overestimate PTP of CAD in a multi-centre study setting with patients being also only referred to CT not ICA [14]. This was shown in our study as well. However, in our patient collective, patients were referred to both, CTA and ICA, based on clinical estimation. The study population of Cheng et al was also characterised by a relatively low prevalence of disease of 15%. The recently published study of Foldyna et al showed overestimation of the D+F model in a large multi-centric cohort from the PROMISE trial [26]. This is in accordance with the results of our study. Again, in comparison with our study, the study of Foldyna et al only included patients being randomised to CTA.

Thus, the analysis of our European DISCHARGE cohort provides a unique opportunity to make comparisons between pre-test models for patients referred for both CTA and ICA in 25 European sites providing performance analysis for both models in a non-selective pragmatic cohort.

### Strengths and limitations

This multi-centre study included patients from clinical sites across Europe. Our patient population is very robust, including patients from 25 different clinical sites, with a high overall patient number of more than 1000 patients, and an almost similar male-to-female ratio in both the CT and ICA group. This pilot study was planned to prepare the subsequent randomised controlled trial. Therefore, patients were routinely sent for CT and ICA examinations per clinical indication. However, this study was not designed as a controlled randomised trial.

In this study, we used both CT and ICA examination as the diagnostic gold standard. This is due to the fact that various clinical studies have proven the high diagnostic accuracy of coronary CT angiography and its high negative predictive value [27]. In our study population, the clinical presentation significantly differed between the CT and ICA group. Due to the design of the D+F model, patient gender and age are part of the PTP calculation and these differences do not reduce the validity of our patient collective, but reflect routine clinical decision-making. In this study, we did not acquire detailed information on the patients’ medical histories (e.g. diabetes, arterial hypertension, smoking status, family history of CAD), CT/ICA indicators of CAD (e.g. coronary calcium score, left ventricular function, myocardial perfusion, wall motion), or adverse events in the follow-up. Thus, we were not able to perform a more detailed analysis to assess the differences in the prevalence of obstructive CAD; we observed between the different clinical sites or using other tests for estimation of pre-test probability (e.g. SCORE or the extended D+F model). As the current European guidelines recommend the D+F model statistically updated by Genders et al in order to select patients for further diagnostic tests, we decided to request only clinical data for the fast and intuitive initial and updated D+F estimation model.

Both the AUCs of the D+F models and the prevalence of significant CAD, which reflects post-test probability, varied strongly among the European sites participating in the study.

## Conclusions

This study demonstrates that the initial and updated D+F models relevantly overestimate the PTP of CAD compared with its actual prevalence in patients routinely selected for both CT and ICA. The updated D+F model was slightly more accurate than the initial D+F version. The prevalence of obstructive CAD differs between clinical sites in Europe. Thus, in order to choose the most beneficial diagnostic test for patients as recommended by the European guidelines, the overestimation of the actual prevalence of CAD and differences in prevalence among European countries need to be considered. More accurate clinical prediction tools are needed to optimize clinical decision-making for the diagnostic management of patients with suspected CAD.

## Electronic supplementary material

Online Figure 1 Distribution of actual disease prevalence and bubble chart of AUCs by clinical site

Twenty-five clinical sites across Europe were involved in the pilot study. The overall CAD prevalence ranged between 15.3% and 49.2%, between 23.3% and 76.7% in the ICA group and between 3.4% and 34.5% in the CT group (see the Appendix for further details). The disease prevalence was significantly higher in the ICA group compared with the CT group (*p* < 0.001).

The bubble chart shows the variability in the relationship between three variables at the aggregate level: diagnostic accuracy (measured by the area under the receiver operating curve AUC), CAD prevalence and mean PTP using the updated D + F model between the 25 clinical sites which recruited patients for the study. The highest AUC are obtained for a wide range of CAD prevalence whereas lower AUC values are obtained in sites where the mean PTP and the CAD prevalence are generally high. The size of the bubble represents the AUC level (small bubble 0.5;0,65, medium bubble 0.65;0.8 and large bubble 0.8;1).

Online Figure 2 ROC examples

The 25 clinical sites which recruited patients for the pilot study showed markedly different AUCs. The AUC for the updated D + F ranged between 0.54 (AUC; 95% CI 0.39-0.70) and 0.91 (AUC; 95% CI 0.82-0.99) for the different clinical sites. The AUCs for the initial D + F version ranged between 0.56 (AUC; 95% CI 0.42-0.71) and 0.90 (AUC; 95% CI 0.84-0.98). This figure shows 3 examples of AUCs for both prediction models to illustrate the variability of discriminative ability between the sites (on the left corresponding site 5 in fig 4, in the middle corresponding site 14 in fig 4, on the right corresponding site 22 in fig 4).

Appendix 1 STROBE checklist

Appendix 2 ROC overview of all 25 clinical sites

Appendix 3 Vendor specific CT protocols for all 25 clinical sites

ESM 1 (DOCX 1137 kb)

## Abbreviations

AUC: The area under the receiver-operating-characteristic curve
CAD: Coronary artery disease
CT: Computed tomography
D+F: Diamond-Forrester
ICA: Invasive coronary angiography
pCRF: Paper-based case report forms
PTP: Pre-test probability
